# 'PhysioDirect' telephone assessment and advice services for physiotherapy: protocol for a pragmatic randomised controlled trial

**DOI:** 10.1186/1472-6963-9-136

**Published:** 2009-08-03

**Authors:** Chris Salisbury, Nadine E Foster, Annette Bishop, Michael Calnan, Jo Coast, Jeanette Hall, Elaine Hay, Sandra Hollinghurst, Cherida Hopper, Sean Grove, Surinder Kaur, Alan Montgomery

**Affiliations:** 1 Academic Unit of Primary Health Care, Department of Community Based Medicine, University of Bristol, 25/27 Belgrave Road, Bristol, BS8 2AA, UK; 2 Arthritis Research Campaign National Primary Care Centre, Primary Care Services, Keele University, Keele, Staffordshire, ST5 5BG, UK; 3 School of Social Policy, Sociology and Social Research, Cornwallis North East, University of Kent, Canterbury, Kent, CT2 7NF, UK; 4 Department of Health Economics, School of Health & Population Sciences, Public Health Building, University of Birmingham, Edgbaston, Birmingham, B15 2TT, UK; 5 Physiotherapy service, Bristol Community Health Physiotherapy Service, Hampton House, Cotham, Bristol, BS6 6AU, UK

## Abstract

**Background:**

Providing timely access to physiotherapy has long been a problem for the National Health Service in the United Kingdom. In an attempt to improve access some physiotherapy services have introduced a new treatment pathway known as PhysioDirect. Physiotherapists offer initial assessment and advice by telephone, supported by computerised algorithms, and patients are sent written self-management and exercise advice by post. They are invited for face-to-face treatment only when necessary. Although several such services have been developed, there is no robust evidence regarding clinical and cost-effectiveness, nor the acceptability of PhysioDirect.

**Methods/Design:**

This protocol describes a multi-centre pragmatic individually randomised trial, with nested qualitative research. The aim is to determine the effectiveness, cost-effectiveness, and acceptability of PhysioDirect compared with usual models of physiotherapy based on patients going onto a waiting list and receiving face-to-face care. PhysioDirect services will be established in four areas in England. Adult patients in these areas with musculoskeletal problems who refer themselves or are referred by a primary care practitioner for physiotherapy will be invited to participate in the trial. About 1875 consenting patients will be randomised in a 2:1 ratio to PhysioDirect or usual care. Data about outcome measures will be collected at baseline and 6 weeks and 6 months after randomisation. The primary outcome is clinical improvement at 6 months; secondary outcomes include cost, waiting times, time lost from work and usual activities, patient satisfaction and preference. The impact of PhysioDirect on patients in different age-groups and with different conditions will also be examined.

Incremental cost-effectiveness will be assessed in terms of quality adjusted life years in relation to cost.

Qualitative methods will be used to explore factors associated with the success or failure of the service, the acceptability of PhysioDirect to patients and staff, and ways in which the service could be improved.

**Discussion:**

It is still relatively unusual to evaluate new forms of service delivery using randomised controlled trials. By combining rigorous trial methods with economic analysis of cost-effectiveness and qualitative research this study will provide robust evidence to inform decisions about the widespread introduction of PhysioDirect services.

**Trial registration:**

Current Controlled Trials ISRCTN55666618

## Background

### The scale of the problem

Musculoskeletal problems are very common, accounting for 15% of all consultations with general practitioners (GPs) in the United Kingdom (UK) [1]. Many of these patients are referred to physiotherapists, with 4.4 million new referrals to physiotherapy being made each year [2]. Ensuring timely access to physiotherapy has long been an issue within the NHS, with waiting times for treatment of several months in some areas. This is a problem for patients, because musculoskeletal conditions cause pain and disability, and for the economy because these conditions are second only to mental health problems as a cause of days lost from work. In particular, back pain accounts for some 120 million days of certified absence from work each year and half of all patients with back pain who are off work for more than 6 months never return to employment [3]. Delayed access to physiotherapy is also a problem for the NHS because when patients are finally offered a physiotherapy appointment many fail to attend, and in other cases patients wait a long time for a physiotherapy consultation when it is unlikely that this will offer benefit, so much of the current physiotherapy resource is used inefficiently and ineffectively.

### PhysioDirect as a new way of improving access to care

In response to these problems, several areas in the UK have introduced a new form of treatment pathway, known as 'PhysioDirect'. In this approach, patients seeking physiotherapy are invited to telephone a physiotherapist for an initial assessment and advice, following which the patient is posted written self-management and exercise advice. Patients are invited for a face-to-face consultation if the initial telephone assessment establishes that this is necessary or if the patient does not get better after following the initial advice.

PhysioDirect is based on a physiotherapist following a computerised algorithm to assess the patient in a structured way, and offering appropriate written advice. This reflects a wider trend to explore the use of this type of technology in health care, exemplified by the development of NHS Direct within the UK. Although there is little evidence about the role of telephone assessment in the context of physiotherapy, research on the use of telephone triage systems in other clinical settings has shown that it is safe, clinically accurate, cost-effective, acceptable to patients, and reduces the workload of clinicians [4-7].

### The effectiveness of physiotherapy

Research about the effectiveness of alternative approaches to provision of physiotherapy services needs to take account of the evidence about the effectiveness of different forms of physiotherapy in different conditions. For some conditions (for example acute low back pain) it appears that manual physiotherapy offers little benefit over simple advice. In conditions such as chronic back pain, promoting exercise appears to be effective [8-11], while systematic reviews about the effectiveness of manipulation have reached inconsistent conclusions [12,13]. Importantly, recent trials have shown that a single session of advice from a physiotherapist is as effective as a course of routine physiotherapy for patients with mild to moderate back problems [14,15] Similarly, several recent trials have shown that physiotherapy-led advice and exercise are effective in knee pain [11,16,17]. On the other hand, for some other conditions such as neck pain and shoulder pain there is evidence that adding manual physiotherapy interventions to advice and exercise is more effective than advice and exercise alone [18-20].

In summary, it is becoming clear that for some conditions patients may benefit from physical treatment such as exercise and manual therapy from a physiotherapist, for other conditions it is equally effective and may be cheaper to provide brief advice about self-management and exercise, and in some situations physical treatments from physiotherapists have little to offer. Therefore the concept of an approach which provides assessment, advice and triage initially and reserves more intensive (and expensive) treatments for those who do not improve may be the most cost-effective strategy. This is analogous to the 'stepped care' approach which is increasingly advocated in a range of conditions, for example mental health, where there is a high level of demand and a need to target resources. In the context of physiotherapy, this approach could reduce costs for patients and for the NHS, provide earlier advice for all patients and physical treatment more quickly for those who may benefit from it (by screening out those unlikely to benefit from it), and be more convenient and accessible for patients as a whole.

### Is faster access beneficial?

The drive to provide faster access to physiotherapy is predicated on the assumption that providing treatment at an earlier stage is beneficial compared with delayed treatment. There is evidence from several studies that early physiotherapy intervention in some situations provides faster symptom relief, improves quality of life, reduces absenteeism, leads to a reduction in physician consultations, and is more cost-effective [21-24].

### Existing evidence about PhysioDirect

Local evaluations and one small trial of PhysioDirect suggest that services based on telephone advice given by physiotherapists are likely to be popular with patients [25], although there is no evidence about costs or outcomes, or the important issue of safety. Audits in the pioneering services in Cheltenham and Huntingdonshire suggested that 40–60% of patients referred by GPs to physiotherapy can be managed by telephone alone without a face-to-face consultation, telephone consultations take approximately half as long as face-to-face consultations, waiting times for a face-to-face appointment were reduced from four weeks to 10 days and 'did-not-attend' (DNA) appointment rates were reduced from 15% to 1%. Patients were very satisfied with the service, with 80% rating it as good or excellent [25-27]. These findings demonstrate the potential benefits of PhysioDirect, but the lack of comparative data from concurrent controls, and a lack of robust data about clinical outcomes and costs, means that there is insufficient evidence currently available to justify the widespread implementation of this new way of working.

### Research questions

This paper describes the protocol for a randomised controlled trial of 'PhysioDirect' versus usual care based on patients going onto a waiting list for a face-to-face appointment.

The research questions are:

• Is PhysioDirect at least as effective as usual models of physiotherapy based on patients going onto a waiting list for face-to-face physiotherapy?

• What is the cost-effectiveness of PhysioDirect compared with usual care?

• Do patients prefer PhysioDirect services rather than usual care; do they find PhysioDirect more convenient and does it address their perceived needs?

• What are the health outcomes and experiences of different groups of patients (those in different age groups and with different types of problems) when referred to PhysioDirect rather than usual care?

## Methods

### Study design

The study consists of an individually randomised pragmatic randomised controlled trial with two parallel groups, incorporating economic evaluation and nested qualitative research. The comparison is between patients randomised to a service based on initial telephone assessment and advice from PhysioDirect, followed by face-to-face treatment when necessary, versus usual care consisting of allocation to a waiting list followed by face-to-face care for all patients.

### Setting

PhysioDirect services will be newly established in four areas of England. Each physiotherapy service will provide care for patients from a defined group of practices within one of the following Primary Care Trusts (PCTs): Bristol, Somerset, Stoke-on-Trent, Central and Eastern Cheshire PCT. The total population covered across all four PCTs will be about 625,000 people. The existing physiotherapy services received about 18,300 referrals from primary care professionals in these practices in 2008.

### Participants

The inclusion criteria are as broad as possible in order to maximise generalisability and to reflect the 'real-world' operation of PhysioDirect services. Inclusion criteria are adults (aged 18 years or over) who are referred by GPs or other members of the primary health care team, or who refer themselves (self-referred) for musculoskeletal physiotherapy.

Exclusion criteria are children (< 18 years); patients referred to physiotherapy by a hospital consultant, emergency department or primary/secondary care interface service; those needing domiciliary physiotherapy; those needing post-operative physiotherapy; those needing physiotherapy for non-musculoskeletal problems; those unable to communicate by telephone in English.

GPs or health care professionals in the relevant practices will refer patients to physiotherapy in their usual way, or patients may refer themselves. Referral forms will be screened by a senior physiotherapist within one working day to confirm that the patient is potentially eligible, and eligible patients will be sent information about the trial by post, along with a consent form and a baseline questionnaire. Patients who give consent to participate and complete the baseline questionnaires will be randomised in a 2:1 ratio to PhysioDirect or usual care. This allocation ratio was chosen to ensure that sufficient people are randomised to PhysioDirect to make it viable to establish this new service, given that non-consenting and excluded patients will continue to receive usual care as well as those randomised to usual care. Randomisation will be undertaken using web or telephone access to a secure remote allocation system, with allocation made at the level of the individual, minimising by PCT, gender, patient age group and presenting complaint. Following randomisation, patients will be sent a letter either inviting them to contact PhysioDirect and telling them how to do so (intervention arm), or telling them that they are on a waiting list for a face-to-face appointment (control arm).

The number of patients excluded from the trial for different reasons will be recorded. The age and sex of all patients will be recorded in anonymised form, to make it possible to compare participating and non-participating populations. Eligible patients who decline to participate will receive usual care.

### Description of intervention and control arms

#### Intervention

'PhysioDirect'. As soon as possible after consent to participate in the trial is received the patient will be invited to telephone a senior physiotherapist for initial assessment and advice. The physiotherapist will follow a computerised algorithm (as developed by the PhysioDirect service in Huntingdonshire) to assess the patient and record findings. In most cases, at the end of the consultation the physiotherapist will post a relevant advice leaflet about self-management and exercises to the patient, inviting them to phone back to report progress after about 2 to 4 weeks if appropriate. At that point they can be given further advice or be booked for a face-to-face consultation if necessary. Alternatively, the initial phone call may establish that more urgent face-to-face care is needed, in which case this will be booked at the outset, or the assessment may establish that physiotherapy is unlikely to be effective and the patient can be given appropriate advice and discharged. In this way those patients most likely to benefit from face-to-face physiotherapy should be able to receive it more quickly, hopefully leading to a faster improvement and a quicker return to work and usual activities.

All four physiotherapy services participating in this trial will set up a PhysioDirect service following the same model of organisation and using the same assessment software, as developed in Huntingdonshire. All physiotherapists operating the PhysioDirect services will undertake a structured training programme and be assessed and certified as competent to undertake PhysioDirect before they assess patients in the trial. Training and assessment of competency will be undertaken by PhysioDirect trainers from Huntingdon PCT. In this way, we will seek to ensure consistency in the way the intervention is developed in each service. Each PhysioDirect service will have a run-in period of at least two months to ensure smooth running of the service before patients to be included in the trial are recruited.

#### Control

Usual care involves patients being referred by a GP or other member of the primary health care team to a physiotherapist. In some areas, patients may also refer themselves directly. Patients go onto a waiting list for an initial face-to-face physiotherapy assessment and then usually have a series of follow-up treatment appointments for several weeks or months. The waiting time may differ considerably at different services, at different sites providing physiotherapy within one service, and at different times of year.

### Outcome assessment

Outcomes will be assessed at baseline, and at 6 weeks and 6 months after randomisation.

The primary outcome is clinical outcome at 6 months, assessed using the physical component summary (PCS) measure from the SF-36v2 questionnaire [28]. The SF-36v2 PCS is a well recognised generic measure of health status. It is particularly suitable for this trial because, unlike disease specific measures, it is applicable to the wide range of musculoskeletal problems referred to physiotherapy.

Two further measures of clinical outcome will be used and considered as important secondary outcomes. The first is the MYMOP2 [29] questionnaire, which is a patient generated measure. It allows patients to specify up to two symptoms and one functional limitation for which they have been referred to physiotherapy, and follow-up questionnaires assess change in those specific symptoms/limitations. This individualised and validated measure can also be used by patients with a wide range of problems [29]. Secondly, a single question will be included as a global measure of individual perception of overall improvement (based on a seven point Likert scale from 'very much worse' to 'very much better'). This type of global improvement score is recommended by the Outcomes Measures in Rheumatology Clinical Trials- Osteoarthritis Research Society International (OMERACT-OARSI) initiative [30].

Other secondary outcomes are:

• costs

• quality of life (measured using the EQ5D measure [31]

• the individual scales and the mental component summary measure from the SF-36

• waiting times for treatment

• time lost from work and usual activities

• satisfaction with care provided

• preference for telephone or face-to-face assessment.

### Collection of data

The baseline questionnaire will collect data about patient characteristics and about the outcome measures. Data about outcomes at follow-up will be collected from postal questionnaires sent at 6 weeks and 6 months after randomisation. Data will be collected by telephone instead of post where patients do not respond to a postal questionnaire or a postal reminder, where patients express a preference for telephone administration, or where patients are unable to complete written questionnaires in English. Data about use of healthcare resources will be extracted from physiotherapy service records and medical records, and details of use of other resources will come from the patient questionnaires.

Table 1 lists the various outcome measures, the timing of data collection and the source of the data.

**Table 1:** Outcome measures, timing of data collection and source of data

	**Timing**	**Source**
Patient identifiers, type of problem, age and sex	Pre-consent	Referral letter. Recorded (anonymised) in research database

Demographic details	Baseline	Baseline patient questionnaire

SF36v2	Baseline, 6 weeks, 6 months	Patient questionnaires

MYMOP	Baseline, 6 weeks, 6 months	Patient questionnaires

EQ5D	Baseline, 6 weeks, 6 months	Patient questionnaires

Overall perception of improvement	6 weeks, 6 months	Patient questionnaires

Time lost from work and usual activities	Baseline, 6 weeks, 6 months	Patient questionnaires

Satisfaction with care provided	6 weeks, 6 months	Patient questionnaire

Preference for Physiodirect or usual care	Baseline, 6 months	Patient questionnaires

Waiting time for treatment	Collected at end of study	Physiotherapy service records, from date of referral received to date first phone or face-to-face consultation

Patient and companion costs	6 weeks, 6 months	Patient questionnaires

Cost of lost production associated with time off work and usual activities.	6 weeks, 6 months	Estimated from information in patient questionnaires

Costs of providing physiotherapy	Set up costs: collected during set up phase and once the service is operating. Treatment over 6 months: collected at end of study.	Set up: data from PCTs about resources involved in setting up the service; Treatment: physiotherapy records, data collected within the trial about lengths of consultations, staff use of time, staff grades etc.

Costs in general practice (consultations, treatments, investigations)	Collected at end of study, from randomisation to 6 months	Patients GP records, consultations costed using Netten and Curtis and NHS reference costs for other costs

Costs of prescriptions	Collected at end of study, from randomisation to 6 months	Patients GP records, costed using British National Formulary

NHS secondary care costs (outpatients, inpatients, admissions)	6 weeks, 6 months	Patient questionnaires for resource use, costed using NHS tariffs

Process evaluation: number, type & duration of consultations with physiotherapists; non-attended appointments with physiotherapists.	Collected throughout study from randomisation to 6 months	Physiotherapy records

Qualifications and experience of physiotherapists.	Collected at the beginning of the study	Online questionnaire completed by physiotherapists

Complaints and adverse events	Collected throughout study from randomisation to 6 months	Notified by patients, physiotherapy services, general practices or any other sources.

### Economic evaluation

The economic evaluation will be carried out from three perspectives. Costs to the NHS will include the cost of providing physiotherapy plus any other costs related to treating the condition for which the patient has been referred to the physiotherapy service. These include: primary care consultations, treatments and investigations, medication, secondary care consultations, and inpatient care. Set-up costs associated with the service will also be collected. Patient and companion costs will include: travel, dependent carer costs, private treatment, over-the-counter medication, and loss of earnings. Societal costs will include use of social services, disability payments, and time off work.

Data will be obtained from four main sources: the patient self-completion questionnaires at baseline, 6 weeks and 6 months after randomisation; primary care patient records; patient records at the physiotherapy services; and records kept as part of the trial detailing length of consultation, grade of staff, and amount of non-contact time. A time and motion study will be conducted to observe activities during non-contact time to estimate the opportunity cost of that time.

Curtis [32] will be used to value primary and community care consultations. Primary care investigations will be valued at cost; the Department of Health tariff will be used for A&E visits, out-patient visits and investigations, and inpatient stays; and the British National Formulary will be used to cost prescribed medication. Most patient and carer costs will be directly reported; the AA schedule for valuing mileage will be used to cost car journeys Time off work by patients and carers will be valued using patient-level information on how absenteeism is dealt with by employers, and will adopt the friction approach, which includes only the resources required to replace the employee [33]. There will be no need to discount costs or outcomes, as they will cover a period of less than one year.

### Process evaluation

Process data about the physiotherapy services provided will include: the number, type and duration of consultations with physiotherapists; rates of non-attended appointments with physiotherapists; and the qualifications and experience of all the physiotherapists involved at all four sites. Complaints and adverse events relating to the physiotherapy services will be systematically recorded and investigated.

### Qualitative research

Qualitative research will be conducted alongside the trial to study issues of implementation and to understand the key barriers and facilitators to the success of a PhysioDirect service. The qualitative research will also examine the acceptability of the service and identify the factors (organisational, professional and patient related) which influence its implementation. The interviews will be conducted in person or by telephone and longitudinal data collection will allow themes to be followed up with participants over time.

Interviews will be carried out with a purposive sample of 40–60 patients to explore the accessibility of the service, the value, acceptability and influence of telephone and face-to-face care, and how services could be improved. Participants in the interviews will be selected to ensure a diverse sample based on age, gender, ethnicity and presenting clinical problem in order to get the widest range of opinions and experiences.

Interviews with a purposive sample of between 20–30 key informants (physiotherapists, GPs, practice-based commissioning leads and managers) will address contextual factors that act to facilitate or hinder the use and implementation of PhysioDirect or usual physiotherapy services, or that influence outcomes, and the perceived value of the services.

### Ethics

Ethical approval for this study has been obtained from Southmead Research Ethics Committee, reference 08/H0102/95.

### Trial registration

Current Controlled Trials ISRCTN55666618

### Analysis

The main hypotheses are that PhysioDirect will be clinically equivalent to usual care and more cost-effective at 6 months after randomisation.

Analysis and presentation of data will be in accordance with CONSORT guidelines. The *primary analysis *will employ multivariable regression to investigate between-group differences in mean SF-36 PCS score at six months follow up. The primary analysis will be conducted on an intention-to-treat (ITT) basis, with due emphasis placed on the confidence interval for the between-arm comparison when inferring equivalence (or otherwise) of the two groups. Clinical equivalence between the arms will only be concluded if the 95% confidence interval for the primary outcome lies wholly inside the range -0.2 to 0.2 SDs. Given the equivalence design and the generally conservative nature of ITT analysis, between-group differences will also be investigated using methods that model compliance with the allocated treatment arm, such as instrumental variable regression. Analyses will adjust for minimisation variables and baseline outcome variable scores, and will take appropriate account of the hierarchical nature of the data (practice and physiotherapy site).

In the *economic analysis *mean cost to the NHS per patient in each arm will be compared with mean gain in quality adjusted life year (QALY) using the EQ5D in order to estimate a cost-utility ratio of incremental cost per QALY gain. Secondary analysis will explore the QALY gain using the SF-6D [34], derived from the SF-36. A cost-consequences matrix will be used to present a comparison of cost per patient in each group from the patient and societal perspectives with a range of outcomes, for example, personal perception of improvement and patient satisfaction. The effect of uncertainty in unit cost estimates or assumptions about resource use will be addressed in sensitivity analyses. Using a bootstrapping approach for data analysis, the results of the economic analysis will be presented using cost-effectiveness acceptability curves.

### Secondary analyses

are summarised in Table 2.

**Table 2:** Secondary analyses:

(1)	Assessing equivalence in clinical outcome using the MYMOP2 score.
(2)	Examining clinical outcome at 6 weeks using the SF-36 PCS

(3)	Comparing the proportion of patients who 'respond to treatment' in each arm, in line with the Outcomes Measures in Rheumatology Clinical Trials- Osteoarthritis Research Society International (OMERACT-OARSI) recommendations [30], using the SF-36 physical function and bodily pain scales and the global improvement score

(4)	Repeating the primary analysis adjusting also for any variables exhibiting marked imbalance at baseline to check that this does not influence the findings

(5)	Analyses as above for secondary outcomes (where p-values will be adjusted to account for multiple testing)

(6)	Investigating the effectiveness and cost-effectiveness of PhysioDirect for patients of different age-groups, or with different presenting problems

(7)	Investigating the effectiveness and cost-effectiveness of PhysioDirect in each of the four PCTs, in exploratory sub-group analyses

(8)	Investigation of process measures such as physiotherapy consultation rates, physiotherapy 'did-not-attend' rates and consultation rates with other health care services in the NHS and private sectors

#### Sub-group analyses

Appropriate interaction terms will be entered into the primary regression analysis for SF-36 in order to conduct pre-specified subgroup analyses according to presenting complaint, patient age-group, socio-economic status and PCT. Since the trial is powered to detect overall equivalence between the groups rather than interactions of this kind, the results of these essentially exploratory analyses will be presented using descriptive statistics and, where helpful, confidence intervals as well as p-values, and interpreted with due caution.

Although the study is powered in relation to patient level outcomes, some of the outcomes will be strongly clustered by physiotherapy site (particularly waiting times). The analysis of these outcomes will be descriptive only.

#### Qualitative analysis

Analysis of the qualitative data will use an inductive, thematic approach based on the method of constant comparison. Data collection and analysis will be carried out iteratively so that emerging themes in the analysis can be explored in depth in further interviews. Sampling will continue until no new themes emerge. A researcher will code the transcribed data, with a sample of interviews being independently coded by a second researcher to ensure transparency and agree emergent themes at successive stages of the data collection and analysis. Negative or deviant cases will be investigated closely.

### Sample size and power calculations

This study is powered to establish clinical equivalence using the PCS scale from the SF-36. The minimum clinically important difference in a range of populations and conditions has been estimated as being at least 4 points (0.4 SD) [35-38]. However in this study a difference of no greater than 2 points has been specified as demonstrating equivalence. This is equivalent to an effect size of 0.2, which is considered a small effect size [38]. Sample sizes for analysis of 488 and 976 in the Usual Care and PhysioDirect groups respectively will yield 80–95% power to reject a null hypothesis of non-equivalence with an overall alpha of 0.05 alpha assuming that the observed difference in means is in the range zero to 0.045 SDs. The target sample size for patients completing the final six-month follow-up questionnaire is 1000 patients in the PhysioDirect arm and 500 patients in the Usual Care arm.

Assuming 20% non-collection of the primary outcomes, it will be necessary to recruit 1250 and 625 patients in the PhysioDirect and usual care arms respectively, or 1875 patients in total.

## Discussion

### The use of a randomised controlled trial to evaluate a service innovation

Changes in the organisation and delivery of health services affect large numbers of people. Relatively small differences in outcome or cost at an individual level can have a big impact at a population level and major financial consequences for the health care system. Despite these considerations, many innovations in health care delivery are implemented based on very limited evidence about costs or effects. This trial represents a relatively unusual example of a large multi-centre randomised controlled trial designed to provide rigorous evidence about the costs and benefits of a new approach to service delivery. Embedding a trial within routine services, in the context of many other pressures on managers and staff, is challenging. The design of the study also raised a number of important methodological issues as described below.

### Level of randomisation

When interventions are designed at the level of an organisation it is often most appropriate to randomise patients in clusters by organisation rather than individually. This is in order to avoid problems of contamination, with patients within the same service receiving different treatments. However for a study of this type, involving a small number of services, cluster randomisation would require a much larger sample size in what is already a large trial. It also raises difficulties in relation to informed consent, and may lead to bias due to differential recruitment rates between patients invited to the intervention or control arms. In this study, it is possible to randomise patients individually with little risk of contamination. However, individual randomisation makes it difficult to assess some outcomes (such as waiting times) which are affected by the existence of two treatment pathways within one organisation. The research team will give particular attention to maintaining separate waiting lists for patients in the PhysioDirect and usual care arms, and allocating equivalent levels of physiotherapy resources to patients in each group, but caution will be needed in interpreting findings about these organisational outcomes.

### Establishing equivalence or difference

Most randomised controlled trials test a hypothesis of a difference between groups by disproving a null hypothesis of no difference. In this trial, it is unlikely that PhysioDirect would provide better clinical outcomes than usual care in the long term. Instead the hypothesis is that PhysioDirect would provide equally good clinical outcomes in the long term, while being more cost-effective, providing a faster recovery and return to work and providing important benefits in secondary outcomes such as patient satisfaction and waiting times. For this reason the study is designed as an equivalence trial (to assess equivalence in the primary outcome of clinical outcome at 6 months) while also assessing difference in cost-effectiveness and the secondary outcomes.

### Appropriate outcome measures

In planning this study, there was much discussion about the most appropriate primary outcome and how this should be measured. It was decided to use clinical outcome as the primary outcome for the reasons described below. However, it could be argued that the main purpose of this type of service is to improve cost-effectiveness, and that effectiveness should not be considered without consideration of cost. Just as it is important to consider whether the extra cost of a new and more effective service can be justified in terms of cost per QALY, it is arguable that a new service such as PhysioDirect may be justifiable even if it is slightly *less *effective than usual care, if it is considerably less expensive, and if the extra cost per QALY of usual care is above the threshold used to determine the acceptability of new treatments.

It was decided to use clinical outcome rather than cost-effectiveness as the primary outcome for several reasons. First, it is clearly important to establish whether a new service is at least as effective as an existing service, and at present (rightly or wrongly) it is conventional to assess whether a new treatment is effective before considering issues of cost-effectiveness. Second, the arguments described above about the application of findings with regard to cost-effectiveness are controversial [39]. Third, there are various methodological issues relating to the use of cost-effectiveness as a primary outcome which remain unresolved or introduce large elements of uncertainty (for example, assumptions needed to calculate sample size).

The traditional trial paradigm which places great emphasis on defining a primary outcome has limitations in relation to the evaluation of health service innovations. In reality, policy decisions about the implementation of new services will be based on taking into account all of the different benefits and costs. Different stakeholder groups such as patients, health service managers or physiotherapists, will place different values on different outcomes (for example, managers may place more value on cost and patients on subjective experience).

Having decided to define clinical outcome as the primary outcome, it was necessary to use a generic health measure given the broad range of clinical conditions managed by physiotherapists. Although there is a concern that generic measures may be less responsive than disease specific measures, the physical functioning and bodily pain scales of the SF-36 (which contribute most of the variation in the PCS summary measure used as the primary outcome in this trial) compare reasonably well with disease specific measures in patients with musculoskeletal problems [40-43]. In addition, two further measures of clinical outcome, one using a patient generated measure, are included as important secondary outcomes to provide further evidence about the equivalence (or otherwise) of the two trial arms.

### The use of mixed methods in evaluating a complex intervention

The introduction of PhysioDirect is a 'complex intervention' with several components and possible 'active ingredients'. In line with MRC guidance [44] the study will combine quantitative, qualitative and economic methods in order to understand how and why, as well as whether, PhysioDirect is effective. Using mixed methods in this way will make it possible to understand factors which act as barriers and facilitators to the implementation of PhysioDirect, and also key contextual factors which determine the success or failure of this intervention.

It is important to note that the comparison between PhysioDirect and usual care is not simply between a telephone consultation versus a face-to-face consultation, nor is it between an early telephone consultation versus a 'waiting list control' i.e. receiving no care. It is between two different care pathways, in one of which (PhysioDirect) patients will receive earlier and more easily accessible advice, mainly (but not entirely) by telephone, and in the other pathway (usual care) patients will wait longer on average for initial assessment which will always be face-to-face, but they may have had some care by the time of first outcome assessment. It is the use of telephone triage and assessment that makes the early provision of advice possible: the issues of telephone based care and the earlier provision of care are inextricably linked and cannot be separated in this pragmatic trial.

## Conclusion

This pragmatic trial and associated qualitative research and economic evaluation will provide robust evidence about the benefits and costs of PhysioDirect services in comparison to usual physiotherapy care. This will inform decisions about the future implementation of these services within the NHS, as well as providing wider lessons for the development of other telephone based triage, assessment and advice services in health care.

## Competing interests

The authors declare that they have no competing interests.

## Authors' contributions

This trial is co-ordinated from the Universities of Bristol and Keele. CS is Chief Investigator and lead investigator for the Bristol site. He conceived and led the design of the study, and wrote the first draft of this paper. NF is lead investigator for the Keele site and contributed to all aspects of study design. AB co-ordinates the implementation of the trial and data collection in the Keele site. MC designed and supervises the qualitative aspects of the study. JC contributes to designing the economic analysis. JH provides advice about implementation of PhysioDirect within PCTs. EH provides advice about study design, particularly in relation to musculoskeletal trials. SH designed the economic component of the study. CH is Trial Manager and co-ordinates all aspects of the study. SG provides clinical advice about the implementation PhysioDirect. SK co-ordinates implementation of the study and data collection in Bristol. AM participated in trial design and is the trial statistician.

## Pre-publication history

The pre-publication history for this paper can be accessed here:


